# Rapid eye movement sleep behavior disorder as a model of early neurodegeneration: challenges for research and clinical diagnosis

**DOI:** 10.3389/fnagi.2026.1961735

**Published:** 2026-10-21

**Authors:** Magdalena Guerra-Crespo, Emmanuel Ortega-Robles, Oscar Arias-Carrión

**Affiliations:** 1 Department of Physiology, Faculty of Medicine, Laboratory of Regenerative Medicine, National Autonomous University of Mexico, Mexico City, Mexico; 2 División de Neurociencias Clínica, Instituto Nacional de Rehabilitación, Mexico City, Mexico; 3 Tecnologico de Monterrey, Escuela de Medicina y Ciencias de la Salud, Mexico City, Mexico

**Keywords:** biomarker development, brainstem networks, prodromal neurodegeneration, REM sleep behavior disorder, REM sleep without atonia, synucleinopathies

## Abstract

Rapid eye movement (REM) sleep behavior disorder (RBD) has evolved from a descriptive parasomnia to a clinically informative model for studying early neurodegenerative processes, particularly within the spectrum of α-synucleinopathies. Defined by the loss of REM sleep atonia and the emergence of dream enactment behaviors, RBD reflects dysfunction of brainstem circuits that normally suppress motor output during dreaming. Longitudinal evidence indicates that many individuals with isolated RBD eventually develop neurodegenerative disease, most commonly Parkinson’s disease, dementia with Lewy bodies, or, less frequently, multiple system atrophy, positioning RBD among the most specific clinical markers of prodromal synucleinopathy currently recognized. Mechanistic studies suggest that RBD arises from instability within distributed REM sleep regulatory networks involving dopaminergic, noradrenergic, serotonergic, and cholinergic systems. This network dysfunction may precede classic motor and cognitive symptoms and can be accompanied by early abnormalities in neurophysiology, imaging, cognition, olfaction, autonomic function, and molecular biomarkers. These findings support a model in which RBD may represent one of the earliest clinically accessible manifestations of a multisystem neurodegenerative process, although anatomical origin, propagation patterns, and phenoconversion trajectories vary across patients. Despite these advances, clinical management remains largely symptomatic, and key challenges persist in diagnosis, risk stratification, prognostic counselling, and therapeutic development. A broader clinical framework is therefore needed, in which RBD is not viewed solely as an isolated sleep disorder but also as a potential marker of early neurodegeneration in appropriately characterized patients. Integrating polysomnography with multimodal biomarkers and network-based models of disease may refine risk stratification and support the development of targeted interventions at earlier stages of neurodegenerative disease.

## 1 Introduction

Rapid eye movement (REM) sleep behavior disorder (RBD) has undergone a profound conceptual transformation over the past two decades. Once regarded as a relatively rare parasomnia characterized by dream enactment and nocturnal motor behaviors, RBD is now increasingly recognized as a clinically accessible and biologically meaningful marker of early neurodegeneration, particularly within the spectrum of α-synucleinopathies (Iranzo et al., 2016; Dauvilliers et al., 2018). This shift reflects a convergence of longitudinal, neurophysiological, imaging, and neuropathological evidence showing that isolated RBD frequently precedes the onset of overt neurological disease, especially Parkinson’s disease, dementia with Lewy bodies, and multiple system atrophy (Dauvilliers et al., 2018; Postuma et al., 2019; Miglis et al., 2021).

In this review, the central discussion of neurodegenerative risk, phenoconversion, biomarker stratification, and prodromal synucleinopathy refers primarily to isolated RBD — also termed idiopathic RBD in earlier literature — defined as video-polysomnography-confirmed RBD occurring in the absence of an established neurodegenerative disorder or another clear secondary cause (Dauvilliers et al., 2018; Postuma et al., 2019; Miglis et al., 2021). The broader term RBD is used when discussing the clinical syndrome, polysomnographic features, REM sleep without atonia, and mechanisms of REM motor control (Boeve et al., 2007; Peever and Fuller, 2017; Dauvilliers et al., 2018). This distinction is important because RBD or RBD-like presentations can also occur in association with medications, particularly antidepressants, narcolepsy, autoimmune or paraneoplastic disorders, structural brainstem lesions, and other neurological conditions, in which the implications for future neurodegenerative disease may differ (Iranzo et al., 2016; Dauvilliers et al., 2018; Howell et al., 2023a).

This emerging framework challenges traditional boundaries between sleep medicine and neurology. RBD is not adequately explained only as a disorder of abnormal behaviors during sleep; rather, it reflects dysfunction of the neural systems that regulate REM sleep atonia, embedded within a broader network of brainstem, subcortical, and cortical circuits (Boeve et al., 2007; Peever and Fuller, 2017; Dauvilliers et al., 2018). These networks overlap with regions that may be vulnerable in early neurodegenerative processes, providing a mechanistic bridge between sleep dysfunction and disease progression. The observation that individuals with RBD can exhibit early abnormalities across multiple domains—including dopaminergic signaling, cortical electrophysiology, cognition, olfaction, and autonomic function—supports the notion that RBD may form part of a distributed prodromal syndrome rather than representing an isolated sleep phenomenon in many patients (Fantini et al., 2003; Gagnon et al., 2009; Iranzo et al., 2010b; Postuma et al., 2010; Miglis et al., 2021).

At a clinical level, this reconceptualization has important implications. RBD offers an opportunity to identify individuals at increased risk of neurodegenerative disease at a stage when neurological symptoms are subtle and disease-modifying intervention may, in principle, be more feasible, although this possibility remains to be demonstrated in clinical trials (Schenck et al., 2013; Postuma et al., 2019; Miglis et al., 2021). However, current approaches remain largely symptomatic, focused on reducing nocturnal behaviors and preventing injuries rather than addressing the underlying neurodegenerative process (Aurora et al., 2010; Howell et al., 2023a). This disconnect highlights a critical gap between diagnosis and disease modification and underscores the need for a more integrated, biomarker-driven strategy that aligns clinical practice with emerging biological insights (Schenck et al., 2013; Miglis et al., 2021).

At a mechanistic level, the pathophysiology of RBD points toward instability within the distributed circuits that generate REM sleep and suppress motor output. Physiological REM atonia depends particularly on pontine glutamatergic neurons and downstream GABAergic and glycinergic inhibitory pathways in the medulla and spinal cord, under broader modulation by cholinergic and monoaminergic systems (Boeve et al., 2007; Peever and Fuller, 2017; Dauvilliers et al., 2018). Disruption of these pathways produces a dissociation between preserved cortical REM activation and inadequate motor suppression. Importantly, this dysfunction can appear before classical motor manifestations of parkinsonism, consistent with models in which neurodegenerative pathology may affect REM-regulatory, autonomic, or peripheral networks before producing overt nigrostriatal motor disease (Braak et al., 2003; Postuma et al., 2019; Berg et al., 2021).

On this basis, a unifying hypothesis can be proposed: isolated RBD may represent one of the earliest and most specific clinically detectable manifestations of a systems-level neurodegenerative process characterized by vulnerability of REM sleep regulatory networks and variable involvement of dopaminergic, limbic, autonomic, and cortical systems over time (Dauvilliers et al., 2018; Postuma et al., 2019; Miglis et al., 2021). Within this framework, REM sleep without atonia is not merely a diagnostic feature but a physiological marker of impaired REM motor control and a potential marker of prodromal disease (Miglis et al., 2021; Cesari et al., 2022). The variability in clinical expression, ranging from isolated RSWA to overt RBD and eventual neurodegenerative disease, may reflect differences in pathological burden, topography, propagation pattern, comorbid pathology, and network resilience (Berg et al., 2021; Miglis et al., 2021; Cesari et al., 2022).

This perspective has translational relevance because isolated RBD provides a clinically identifiable population for biomarker development, longitudinal risk stratification, and future disease-modification trials (Schenck et al., 2013; Postuma et al., 2019; Miglis et al., 2021). The following sections build on this framework by integrating clinical, mechanistic, and translational evidence to examine RBD as a model of early brain vulnerability and as a platform for developing biomarkers and potential disease-modifying strategies.

## 2 Methods

To inform this narrative review, we conducted a structured but non-systematic literature search of the PubMed and Scopus databases covering the period from January 1986, when REM sleep behavior disorder was first formally described, to August 2026. Search terms included combinations of “REM sleep behavior disorder,” “RBD,” “isolated RBD,” “idiopathic RBD,” “REM sleep without atonia,” “RSWA,” “polysomnography,” “REM sleep atonia,” “brainstem,” “neural circuitry,” “α-synuclein,” “synucleinopathy,” “Parkinson’s disease,” “dementia with Lewy bodies,” “multiple system atrophy,” “neurodegeneration,” “prodromal,” “phenoconversion,” “biomarkers,” “DAT-SPECT,” “PET,” “EEG,” “cognition,” “olfaction,” and “autonomic dysfunction.” As appropriate for a narrative review, study selection and database filtration were performed at the authors’ discretion according to relevance, methodological quality, mechanistic or clinical significance, and consistency with the conceptual focus of the manuscript. We prioritized original longitudinal and prospective cohort studies, polysomnographic and neurophysiological investigations, molecular and functional neuroimaging studies, neuropathological and mechanistic studies, and consensus statements or clinical guidelines that contributed to the understanding of RBD diagnosis, REM-atonia circuitry, its relationship with neurodegenerative disorders, and biomarkers associated with future phenoconversion. High-quality reviews and meta-analyses were additionally used to contextualize areas with heterogeneous or limited primary evidence. Studies addressing secondary RBD, isolated RSWA, focal brainstem lesions, narcolepsy, autoimmune disorders, or non-synuclein neurodegenerative diseases were included when they contributed to differential diagnosis or to the understanding of REM-sleep motor control and network dysfunction. Studies were generally excluded when RBD or RSWA was not specifically assessed, diagnostic characterization was insufficient, findings were not relevant to the mechanistic, biomarker, or clinical framework of the review, or they provided redundant evidence without additional conceptual or methodological contribution. Isolated case reports were considered selectively when they provided unique mechanistic or clinicopathological information, particularly for rare disorders or focal lesions. Reference lists of key articles, consensus documents, and major reviews were also examined to identify influential or foundational studies relevant to the themes developed in this review. Because the objective was conceptual synthesis rather than systematic evidence grading, no formal meta-analysis or quantitative assessment of study quality was performed, screening was not performed independently in duplicate, and no formal risk-of-bias scoring tool was applied.

## 3 Clinical description

REM sleep behavior disorder is traditionally classified as a parasomnia, defined by the predominance of undesirable motor events during sleep (Boeve et al., 2007; Iranzo et al., 2016; Dauvilliers et al., 2018). This definition remains clinically useful, but it does not fully capture the biological significance of the disorder. RBD is characterized by the loss of physiological REM sleep atonia, resulting in abnormal motor behaviors that reflect the enactment of dream content (Boeve et al., 2007; Peever and Fuller, 2017; Dauvilliers et al., 2018). These behaviors range from simple manifestations, such as talking, laughing, shouting, or brief limb movements, to complex and often violent actions, including gesturing, reaching, grabbing, punching, kicking, or leaving the bed (Iranzo et al., 2005; Dauvilliers et al., 2018). Notably, dream content is frequently defensive or threat-related, despite preserved behavioral control during wakefulness (Fantini et al., 2005; Iranzo et al., 2016).

The clinical burden is considerable. Patients often experience sleep fragmentation and are at high risk of injury, including bruises, lacerations, fractures, and, in severe cases, intracranial trauma (Olson et al., 2000; McCarter et al., 2014; Iranzo et al., 2016). Bed partners are frequently affected, and sleep-related injury remains the most common reason for medical consultation. RBD predominantly affects individuals over the age of 50 years and has been reported more frequently in men; however, this apparent sex difference is likely influenced by referral patterns and differences in symptom expression rather than a purely biological predisposition (Olson et al., 2000; Zhou et al., 2015; Haba-Rubio et al., 2018). Contemporary epidemiological studies using polysomnography estimate a prevalence below 1% in older populations, although underdiagnosis remains a major limitation (Iranzo et al., 2016; Pujol et al., 2017; Haba-Rubio et al., 2018).

## 4 Idiopathic versus secondary RBD

RBD may occur in isolation—historically termed “idiopathic”—or in association with neurological or systemic disorders. This distinction, although still used in clinical practice, requires re-evaluation (Dauvilliers et al., 2018). Longitudinal studies over the past decade have demonstrated that most individuals with idiopathic RBD eventually develop neurodegenerative disease, particularly α-synucleinopathies such as Parkinson’s disease, dementia with Lewy bodies, or multiple system atrophy. Consequently, idiopathic RBD is now widely recognized as a prodromal stage of neurodegeneration rather than a benign condition (Iranzo et al., 2016; Postuma et al., 2019). This shift has profound implications, as it positions RBD as one of the most specific clinical markers of early neurodegenerative disease and a critical entry point for disease-modifying interventions (Schenck et al., 2013).

Secondary RBD has been reported across a broad spectrum of neurological disorders, including Machado–Joseph disease, amyotrophic lateral sclerosis, epilepsy, multiple sclerosis, and Guillain–Barré syndrome, as well as in neurodevelopmental conditions such as autism and Tourette syndrome (Keir and Breen, 2020). These associations suggest that RBD reflects dysfunction within distributed REM sleep regulatory networks rather than a disease-specific mechanism (Boeve et al., 2007). RBD is also commonly observed in narcolepsy, a disorder characterized by hypocretin deficiency and REM sleep instability. In this context, the loss of hypothalamic hypocretinergic input to brainstem nuclei involved in REM atonia—such as the locus coeruleus and subcoeruleus complex—may contribute to motor disinhibition during REM sleep (Dauvilliers et al., 2013). The review by Keir and Breen specifically documents reports involving epilepsy, spinocerebellar ataxia—particularly SCA3/Machado–Joseph disease—amyotrophic lateral sclerosis, Guillain–Barré syndrome, autism, and Tourette syndrome.

Recent work has further expanded the spectrum of RBD by implicating immune-mediated mechanisms. Cases associated with autoimmune limbic encephalitis, including those linked to voltage-gated potassium channel antibodies, have shown reversibility following immunotherapy, indicating that RBD can arise from transient network dysfunction (Cornelius et al., 2011). These observations challenge a purely degenerative framework and highlight the need to consider RBD within a broader systems-neuroscience perspective. In the Cornelius et al. series, dream-enactment behavior was found in patients with limbic encephalitis and Morvan syndrome associated with VGKC-complex autoimmunity, and it resolved completely or almost completely in most treated patients with available follow-up.

Pharmacological factors also play an important role. Antidepressants, particularly serotonergic and noradrenergic agents, can induce or exacerbate REM sleep without atonia and precipitate dream enactment behaviors (Postuma et al., 2013). Although these drugs generally suppress REM sleep, their effects on REM atonia appear complex and incompletely understood (Winkelman and James, 2004). Similar phenomena have been reported with certain beta-blockers and during alcohol withdrawal (Barone, 2024). These findings underscore the sensitivity of REM sleep circuits to neurochemical modulation and have direct clinical implications for medication management in patients at risk of RBD or neurodegeneration. Antidepressant-associated RBD may represent a direct pharmacological effect, the unmasking of latent RBD, or an earlier clinical presentation of an underlying prodromal process.

## 5 Polysomnographic features

A defining feature of REM sleep is the near-complete suppression of skeletal muscle activity. In RBD, this mechanism fails, resulting in REM sleep without atonia (RSWA), which is detected as increased tonic or phasic electromyographic activity during REM sleep. RSWA, in combination with abnormal motor behaviors captured on video-polysomnography, constitutes the electrophysiological hallmark of RBD (Cesari et al., 2022).

Polysomnography with audiovisual recording is therefore essential for diagnosis (Cesari et al., 2022). It allows precise temporal correlation between abnormal behaviors and REM sleep and enables differentiation from mimics such as obstructive sleep apnea, nocturnal seizures, and non-REM parasomnias (Antelmi et al., 2021). Importantly, polysomnography can also identify subclinical phenotypes, including individuals with RSWA in the absence of overt behavioral symptoms (Cesari et al., 2022). These cases may represent an early stage of disease, although their natural history remains incompletely defined and warrants longitudinal investigation (Stefani et al., 2015).

## 6 Diagnostic criteria

RBD was first recognized as a distinct clinical entity in the 1980s and subsequently incorporated into formal classification systems (Schenck et al., 1986; Högl et al., 2022). Early diagnostic criteria relied primarily on clinical features, including dream enactment and injurious behavior (Högl et al., 2022). However, advances in sleep neurophysiology and the recognition of diagnostic mimics have led to a refinement of these criteria (Antelmi et al., 2021). Current standards require polysomnographic confirmation of REM sleep without atonia, alongside documentation of abnormal behaviors during REM sleep and exclusion of alternative causes (Cesari et al., 2022).

This evolution reflects a broader paradigm shift toward precision medicine. Polysomnographic criteria provide greater sensitivity and specificity than clinical assessment alone, enabling more accurate diagnosis and facilitating the identification of individuals at risk of neurodegeneration (Cesari et al., 2022). Moreover, the detection of subclinical abnormalities expands the diagnostic spectrum and creates opportunities for early intervention (Miglis et al., 2021).

## 7 Prevalence of RBD

The prevalence of RBD in the general population remains difficult to establish, largely due to the need for polysomnographic confirmation. Early population-based surveys suggested a prevalence of approximately 0.4–0.5%, whereas subsequent polysomnography-based studies in middle-aged and older populations have reported estimates ranging from approximately 0.7 to 2% (Ohayon et al., 1997; Chiu et al., 2000; Pujol et al., 2017). Importantly, a significant proportion of affected individuals had experienced sleep-related injury without prior diagnosis, highlighting persistent under-recognition (Chiu et al., 2000).

These findings emphasize the need for increased awareness among clinicians and improved screening strategies in at-risk populations. More fundamentally, the recognition of RBD as a prodromal marker of neurodegenerative disease necessitates a shift in clinical practice, from symptomatic management to proactive identification, risk stratification, and integration into longitudinal care pathways (Miglis et al., 2021). For neuroscientists, RBD provides a unique window into the earliest stages of neurodegeneration, offering an opportunity to investigate disease mechanisms and test neuroprotective strategies before irreversible neuronal loss occurs (Schenck et al., 2013).

## 8 Pathophysiology

Experimental models have provided a foundational framework for understanding the neurobiology of REM sleep and its disruption in RBD. Electrophysiological, lesion-based, and neuropharmacological studies in animals consistently show that the generation and maintenance of muscle atonia during REM sleep depend on coordinated activity within a distributed brainstem network. Key structures include the sublaterodorsal (or subcoeruleus) region, the magnocellular and gigantocellular reticular nuclei, the locus coeruleus complex, the dorsal raphe nucleus, and the pedunculopontine tegmental nucleus. These nuclei interact through excitatory and inhibitory pathways to actively suppress spinal motor neuron output during REM sleep. Importantly, these brainstem circuits are not functionally isolated; they maintain reciprocal connections with the basal ganglia and limbic system, linking REM motor control with emotional and motivational processing. Lesion studies further demonstrate that the phenotype of REM-related motor activity depends on the anatomical location and extent of disruption, supporting the concept that RBD reflects network-level disinhibition rather than focal damage (Boeve et al., 2007; Peever and Fuller, 2017). The principal components of the REM-atonia circuitry and their disruption in RBD are illustrated in Figure 1.

**Figure 1:**
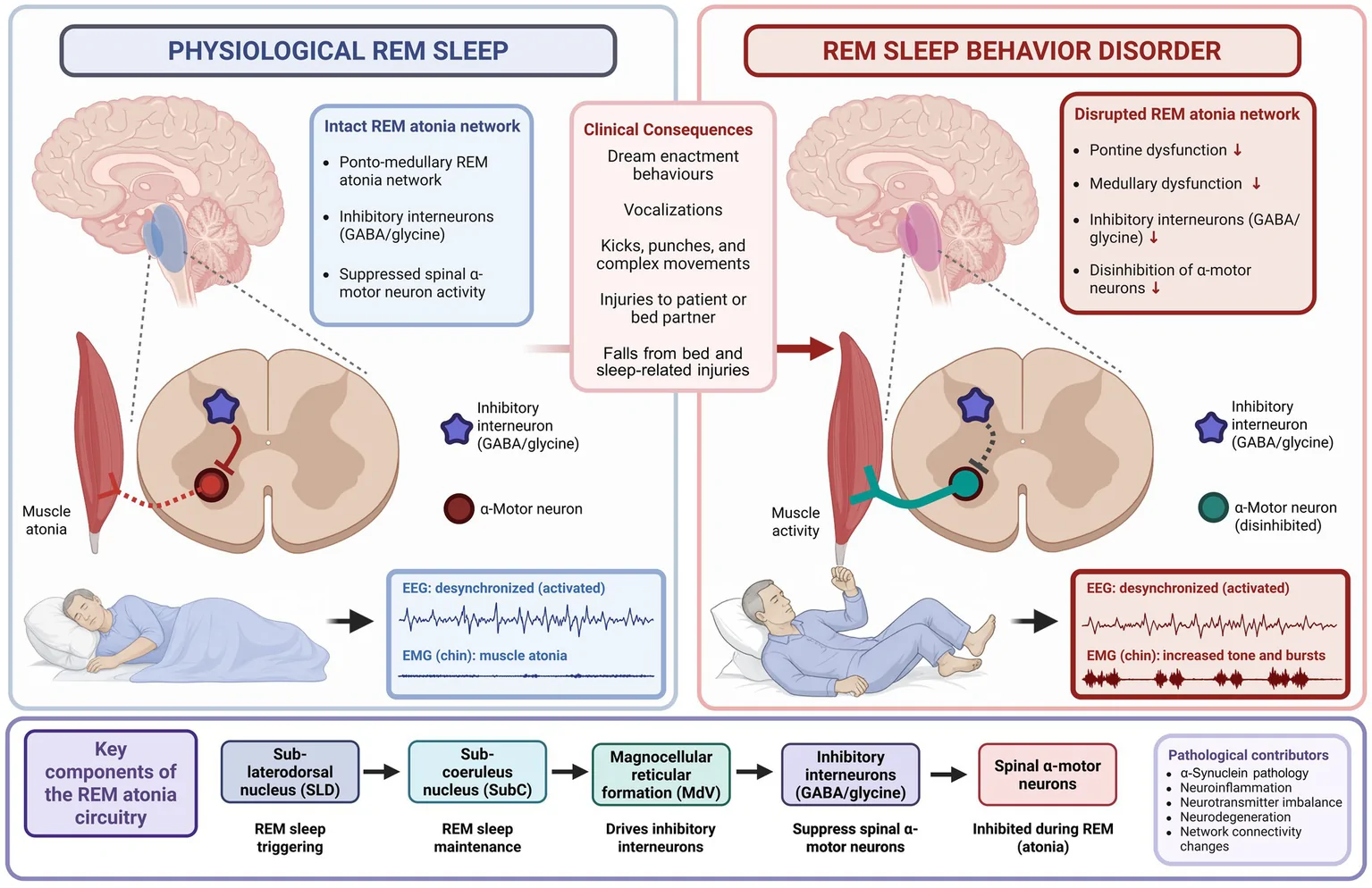
Neural circuitry underlying physiological REM sleep atonia and its disruption in REM sleep behavior disorder. Left panel (physiological REM sleep): The pontomedullary network involved in physiological REM sleep atonia is illustrated. During normal REM sleep, pontine and medullary circuits recruit GABAergic and glycinergic inhibitory pathways that suppress spinal α-motor neuron activity, producing skeletal muscle atonia despite preserved cortical activation. Schematic EEG and chin EMG traces show REM-related EEG desynchronization accompanied by minimal muscle activity. Right panel (REM sleep behavior disorder): Dysfunction within components of the REM-atonia network may reduce inhibitory drive to spinal α-motor neurons. This motor-neuron disinhibition, in the context of preserved REM cortical activation, is associated with tonic and phasic muscle activity during REM sleep and can clinically manifest as dream-enactment behaviors, vocalizations, complex movements, and sleep-related injuries. Lower panel: A simplified organization of the principal components and functional relationships within the REM-atonia circuitry is shown, including the sublaterodorsal nucleus (SLD), subcoeruleus nucleus (SubC), magnocellular reticular formation (MdV), GABAergic and glycinergic inhibitory neurons, and spinal α-motor neurons. Potential contributors to disruption of this network include α-synuclein pathology, neuroinflammation, neurotransmitter imbalance, neurodegeneration, and altered network connectivity. Image created by the authors using BioRender.

In humans, emerging evidence suggests that distinct physiological mechanisms may underlie different components of REM sleep dysregulation. RSWA and excessive phasic motor activity can occur independently, indicating partial dissociation within the REM atonia circuitry. Pharmacological observations support this distinction. For example, clonazepam—widely used in clinical practice—can reduce phasic motor events without fully restoring tonic atonia, implying that inhibitory control over phasic and tonic motor outputs may involve separable pathways. However, these findings remain based on small cohorts and require validation in larger, mechanistically informed studies (Lapierre and Montplaisir, 1992).

Neuropathological investigations have provided critical insights into the human substrate of RBD. Autopsy series consistently demonstrate abnormalities within brainstem regions that regulate REM atonia, particularly the locus coeruleus–subcoeruleus complex and the substantia nigra. These changes include neuronal loss, gliosis, depigmentation, and the presence of Lewy bodies, especially in cases associated with α-synucleinopathies. Additional, though less consistent, involvement has been reported in the dorsal raphe nucleus, dorsal motor nucleus of the vagus, gigantocellular reticular formation, and pedunculopontine nucleus (Boeve et al., 2007). Importantly, the distribution of these lesions overlaps with early Braak stages of Parkinson’s disease, supporting the hypothesis that RBD represents an early manifestation of brainstem-predominant neurodegeneration (Braak et al., 2003; Boeve et al., 2007; Postuma et al., 2019).

Recent post-mortem evidence has further strengthened the biological interpretation of isolated RBD as an early α-synucleinopathy. In a clinicopathological series of 20 individuals with video-polysomnography-confirmed idiopathic RBD, post-mortem diagnoses included Lewy body disease in 19 cases and multiple system atrophy in one case. α-Synuclein deposits were identified in structures involved in REM sleep atonia, including the subcoeruleus nucleus, gigantocellular reticular nucleus, laterodorsal tegmentum, and amygdala (Mayà et al., 2024). Importantly, individuals who remained neurologically unimpaired or developed Parkinson’s disease without dementia showed predominantly brainstem and limbic α-synuclein pathology, whereas those who developed Parkinson’s disease dementia or dementia with Lewy bodies had more diffuse cortical involvement and more frequent comorbid pathologies, especially Alzheimer-type pathology (Mayà et al., 2024). This supports the view that isolated RBD is usually an early α-synucleinopathy, while also emphasizing that clinical heterogeneity may reflect differences in pathological burden, distribution, and co-pathology.

Conventional structural neuroimaging has generally been insensitive to these changes, with most patients showing no abnormalities on standard MRI (Campabadal et al., 2021). Nevertheless, focal lesions affecting the pontomesencephalic region—whether vascular, neoplastic, or surgical, have been reported to induce RBD, providing causal evidence that disruption of REM atonia circuits is sufficient to generate the syndrome. More sensitive techniques, including diffusion tensor imaging and functional imaging, have begun to reveal subtle alterations in brainstem integrity and connectivity in idiopathic RBD, although these findings remain heterogeneous and require standardization (Iranzo et al., 2014; Boucetta et al., 2016; Campabadal et al., 2021).

Molecular imaging has provided some of the most compelling evidence linking RBD to neurodegenerative processes. Single-photon emission computed tomography (SPECT) and positron emission tomography (PET) studies have consistently demonstrated reduced striatal dopamine transporter binding and decreased presynaptic dopaminergic terminal density in patients with RBD, even in the absence of overt motor symptoms (Miglis et al., 2021). These changes appear to follow a continuum, with progressive reductions observed from subclinical RSWA to clinically manifest RBD and, ultimately, Parkinson’s disease (Kunz et al., 2023). Such findings strongly support the concept that nigrostriatal dysfunction begins well before the clinical onset of parkinsonism (Iranzo et al., 2010b; Postuma et al., 2019). However, whether dopaminergic impairment is a primary driver of RBD or a downstream marker of broader neurodegenerative processes remains unresolved.

Other neurotransmitter systems are likely to play complementary roles. Noradrenergic neurons in the locus coeruleus, serotonergic projections from the raphe nuclei, and cholinergic pathways from the pedunculopontine nucleus all contribute to REM sleep regulation and motor inhibition. Disruption across these systems may produce a state of network instability in which REM sleep is preserved at the cortical level but fails to inhibit motor output at the spinal level. This model aligns with recent systems neuroscience approaches, which conceptualize REM sleep as a dynamic balance between excitatory and inhibitory brainstem circuits rather than a unidirectional process (Peever and Fuller, 2017).

In summary, although important mechanistic details remain to be clarified, the pathophysiology of RBD is best understood as a disorder of distributed brainstem and subcortical networks that regulate REM sleep atonia. Dysfunction across dopaminergic, noradrenergic, serotonergic, and cholinergic systems converges to produce motor disinhibition during REM sleep (Dauvilliers et al., 2018). Crucially, this network vulnerability appears to precede and predict the development of neurodegenerative disease in many cases (Postuma et al., 2019). These insights demand a shift in perspective: from viewing RBD as a symptomatic sleep disorder to recognizing it as an early systems-level manifestation of neurodegeneration. This paradigm has direct translational implications, highlighting the need for biomarker-driven diagnosis, longitudinal monitoring, and the development of targeted interventions aimed at modifying disease trajectories at their earliest stages (Miglis et al., 2021).

## 9 Treatment

Despite the clinical relevance and growing recognition of RBD as a prodromal neurodegenerative condition, therapeutic strategies remain largely symptomatic and are supported by limited high-quality evidence. To date, no randomized, double-blind, placebo-controlled trials have definitively established the efficacy of any pharmacological intervention for RBD (Howell et al., 2023b; Howell et al., 2023a). This gap highlights a critical unmet need, particularly in light of the disorder’s prognostic implications and its potential role as a therapeutic window for disease modification (Schenck et al., 2013).

Clonazepam, administered at doses typically ranging from 0.5 to 2 mg at bedtime, remains the most widely used and historically preferred treatment (Aurora et al., 2010). Observational studies and large clinical series consistently report substantial reductions in dream enactment behaviors and associated injuries (McCarter et al., 2013). The therapeutic effect appears to be mediated through suppression of phasic motor activity during REM sleep rather than restoration of physiological atonia. However, clonazepam does not normalize the underlying electrophysiological abnormality, REM sleep without atonia, indicating that it acts primarily at a symptomatic level (Lapierre and Montplaisir, 1992). Its use is further limited by adverse effects, including daytime somnolence, cognitive impairment, gait instability, and exacerbation of obstructive sleep apnea, which are particularly relevant in older populations. Notably, approximately 10% of patients show little or no clinical response, underscoring variability in treatment efficacy and the need for alternative approaches (Aurora et al., 2010; McCarter et al., 2013).

Melatonin has emerged as a clinically valuable alternative, particularly in patients in whom benzodiazepines are contraindicated. Several small studies and clinical observations suggest that melatonin can reduce the frequency and severity of REM-related motor behaviors, with a more favorable safety profile. Its mechanism of action is not fully understood but may involve modulation of circadian rhythms and stabilization of REM sleep architecture. In contrast to clonazepam, melatonin is less likely to impair cognition or exacerbate respiratory disorders, making it especially suitable for patients with comorbid neurodegenerative disease, cognitive decline, or sleep-disordered breathing (Kunz and Mahlberg, 2010; McGrane et al., 2015).

The role of other pharmacological agents remains uncertain. Cholinesterase inhibitors, such as donepezil and rivastigmine, have shown mixed results. Some studies report modest improvements in RBD symptoms, particularly in patients with dementia with Lewy bodies, possibly reflecting enhanced cholinergic modulation of REM sleep circuits (Massironi et al., 2003). However, these findings are inconsistent and not supported by robust controlled trials. Similarly, dopaminergic therapies, including dopamine agonists such as pramipexole, have yielded conflicting results. While some reports describe reductions in motor behaviors during REM sleep, others fail to demonstrate meaningful clinical benefit. These discrepancies likely reflect heterogeneity in underlying pathophysiology, disease stage, and study design (Postuma et al., 2019; Howell et al., 2023b). The AASM evidence review identified only small observational studies and pilot trials for these agents; its corresponding guideline offers conditional recommendations for pramipexole and transdermal rivastigmine in selected clinical contexts (Howell et al., 2023a).

Beyond pharmacological treatment, non-pharmacological strategies are essential and often underemphasized. Environmental modifications, such as removing hazardous objects, padding the sleep environment, or using bed barriers, are critical to reduce injury risk and should be considered a first-line intervention in all patients. Patient and caregiver education is equally important, particularly in the context of the evolving understanding of RBD as a marker of neurodegeneration (Howell et al., 2023a).

In summary, current treatment approaches for RBD remain symptomatic and are supported by limited evidence. Clonazepam continues to be the most effective agent for reducing behavioral manifestations, whereas melatonin offers a safer alternative in vulnerable populations. However, neither therapy addresses the underlying neurobiological mechanisms or alters disease progression. This limitation reinforces the need for a paradigm shift, from managing nocturnal behaviors to developing mechanism-based and potentially disease-modifying interventions. As RBD is increasingly recognized as an early manifestation of neurodegenerative disease, future therapeutic strategies must integrate biomarker-driven risk stratification and target the underlying pathophysiology at its earliest stages.

## 10 RBD and neurodegenerative disorders

### 10.1 RBD in synucleinopathies

RBD is strongly associated with neurological diseases, with earlier series reporting co-occurrence in approximately 38–75% of cases (Gagnon et al., 2006b). Contemporary longitudinal cohorts have refined this estimate, showing that the majority of individuals with idiopathic RBD eventually develop a neurodegenerative disorder, most commonly an α-synucleinopathy (Iranzo et al., 2016; Postuma et al., 2019). These include Parkinson’s disease, multiple system atrophy, and dementia with Lewy bodies, conditions unified by intraneuronal aggregation of α-synuclein and early involvement of brainstem nuclei regulating REM sleep. This convergence has reshaped the conceptual framework of RBD: rather than a coincidental comorbidity, it is now regarded as an early clinical expression of synuclein-driven neurodegeneration (Dauvilliers et al., 2018).

### 10.2 RBD in Parkinson’s disease

Abnormal REM sleep motor activity in Parkinson’s disease was first described several decades ago, but systematic investigations have clarified its prevalence and clinical significance (Dauvilliers et al., 2018). Clinical features of RBD are reported in approximately 15–40% of patients with Parkinson’s disease, whereas polysomnography reveals even higher rates when subclinical phenotypes are included. RSWA, with or without overt behavioral manifestations, may be present in more than half of patients, indicating that REM sleep dysregulation is a common and under-recognized feature of the disease (Gagnon et al., 2002). In the study by Gagnon et al. (2002), 33% of patients with Parkinson’s disease met criteria for RBD, whereas 58% showed RSWA, directly supporting the distinction between clinically manifest RBD and subclinical REM motor dysregulation.

The temporal relationship between RBD and Parkinson’s disease is heterogeneous. In many cases, isolated RBD precedes the onset of motor symptoms by years or even decades, consistent with prodromal disease. In others, it emerges concurrently or after diagnosis, reflecting progression of neurodegeneration (Dauvilliers et al., 2018; Baumann-Vogel et al., 2020). Notably, the presence of RBD does not correlate consistently with motor severity, dopaminergic treatment, or disease duration (Kim et al., 2018). However, accumulating evidence suggests that RBD identifies a distinct clinical phenotype characterized by more diffuse neurodegeneration, with increased risk of cognitive decline, autonomic dysfunction, and neuropsychiatric complications, including hallucinations. These observations reinforce the need to stratify patients with Parkinson’s disease according to sleep phenotypes, as this may have prognostic and therapeutic implications (Kim et al., 2018). The systematic review found that RBD predicted dementia and hallucinations in longitudinal studies and was associated with autonomic dysfunction and a more extensive pathological phenotype in cross-sectional, imaging, and neuropathological evidence.

### 10.3 RBD in multiple system atrophy

The association between RBD and multiple system atrophy (MSA) is particularly robust. Polysomnographic studies indicate that RBD or RSWA is present in nearly all patients with MSA, often early in the disease course (Plazzi et al., 1997). This high prevalence likely reflects the anatomical distribution of neurodegeneration, which in MSA frequently involves pontine and medullary structures critical for REM sleep atonia. Compared with Parkinson’s disease, the more widespread and severe involvement of brainstem circuits in MSA provides a plausible explanation for the near-universal presence of REM sleep motor dysregulation (Ghorayeb et al., 2002).

Interestingly, RBD has also been reported in related synucleinopathies such as pure autonomic failure, further supporting the concept that REM sleep disruption is a core feature of synuclein-driven disease rather than a condition-specific phenomenon (Miglis et al., 2017). From a clinical perspective, the early presence of RBD in MSA may aid in differential diagnosis, particularly in patients presenting with autonomic dysfunction or atypical parkinsonism (Wenning et al., 2022).

### 10.4 RBD in dementia with Lewy bodies

A strong association between RBD and dementia with Lewy bodies (DLB) has been consistently reported, and RBD is now included as a suggestive diagnostic feature in current clinical criteria (McKeith et al., 2017). In many patients, isolated RBD precedes the onset of cognitive impairment, providing an important clue to underlying Lewy body pathology (McKeith et al., 2017). In cases of diagnostic uncertainty—particularly when distinguishing DLB from Alzheimer’s disease, polysomnographic confirmation of RBD can substantially increase diagnostic confidence (Ferman et al., 2011).

Recent studies have further demonstrated that patients with DLB and RBD exhibit more pronounced brainstem and limbic involvement, supporting the notion that REM sleep dysregulation reflects early and widespread network disruption (Dugger et al., 2012; Iaccarino et al., 2016). As in Parkinson’s disease, the presence of RBD may therefore define a biologically distinct and clinically relevant subtype of disease (Murray et al., 2013; Iaccarino et al., 2016).

### 10.5 RBD outside classical synucleinopathies and in mixed-pathology contexts

Although RBD is most strongly associated with α-synucleinopathies, REM sleep without atonia and, less commonly, clinically manifest RBD have also been reported in other neurological conditions. These observations are important for differential diagnosis, but they should be interpreted cautiously because many reports involve small series, limited polysomnographic characterization, or incomplete neuropathological confirmation.

RBD has been described in parkinsonism associated with parkin mutations, a condition traditionally considered less consistently linked to Lewy body pathology. These observations initially challenged the specificity of the RBD–synucleinopathy association; however, subsequent neuropathological evidence showing Lewy body pathology in some families with parkin mutations suggests that overlapping mechanisms or mixed pathology may contribute in selected cases (Kumru et al., 2004; Pramstaller et al., 2005). More broadly, these findings indicate that RBD reflects dysfunction of REM sleep regulatory circuits, even though its strongest and most clinically relevant association remains with synucleinopathies (Boeve et al., 2007).

RBD and RSWA have also been reported in tauopathies, including progressive supranuclear palsy, corticobasal degeneration, and Alzheimer’s disease, but clinically overt RBD appears uncommon in these disorders and may often reflect mixed or coexisting α-synuclein pathology rather than isolated tau or Alzheimer pathology (Arnulf et al., 2005; Gagnon et al., 2006a; Cooper and Josephs, 2009; Boeve et al., 2013; Cullinane et al., 2022). In Alzheimer’s disease, polysomnographically confirmed RBD is rare, and clinicopathological studies frequently identify concomitant Lewy body pathology, suggesting that “pure” Alzheimer pathology is unlikely to be a common independent cause of RBD (Boeve et al., 2013). Similarly, evidence in corticobasal degeneration and Pick’s disease remains limited and inconclusive.

These less common associations emphasize two complementary points. First, RBD is not pathognomonic for α-synucleinopathy and should be interpreted in the context of clinical phenotype, medication exposure, polysomnographic findings, and biomarker data. Second, when RBD occurs in disorders not classically defined as synucleinopathies, mixed pathology or selective involvement of REM-atonia circuits should be considered. Recent post-mortem data further support this view, showing that idiopathic RBD is usually associated with Lewy body disease or multiple system atrophy, while also demonstrating that coexisting Alzheimer-type, tau-related, vascular, and TDP-43 pathologies are frequent in older patients and may influence clinical expression, especially cognitive outcomes (Mayà et al., 2024).

### 10.6 Synthesis and implications

Collectively, the evidence indicates that RBD is most strongly associated with synucleinopathies, particularly Parkinson’s disease, multiple system atrophy, and dementia with Lewy bodies. Although REM sleep abnormalities can occur in other neurodegenerative disorders, clinically overt RBD remains uncommon outside this group and is often attributable to mixed pathology. This pattern likely reflects the preferential involvement of lower brainstem structures—central to REM atonia, in synucleinopathies (Table 1).

**Table 1:** Association of REM sleep behavior disorder with major neurodegenerative disorders.

Disorder	Strength of association with RBD	Typical relationship with RBD	Clinical significance	Reference
PD	Strong	RBD may precede motor symptoms by years or decades, although it may also develop after diagnosis	Identifies a diffuse disease phenotype associated with increased cognitive, autonomic and neuropsychiatric involvement	Dauvilliers et al. (2018) and Kim et al. (2018)
MSA	Very strong	RBD or RSWA is present in most patients, often early in the disease course	Supports the diagnosis of MSA and reflects extensive brainstem involvement	Palma et al. (2015) and Wenning et al. (2022)
DLB	Strong	Frequently precedes cognitive decline	Included as a supportive diagnostic feature; increases diagnostic confidence and reflects early Lewy body pathology	McKeith et al. (2017)
PAF	Strong	May precede conversion to other synucleinopathies	Supports the concept that REM sleep dysregulation is a core feature of α-synucleinopathies	Giannini et al. (2018)
PSP	Limited	RSWA may occur; clinically overt RBD is uncommon	May reflect brainstem involvement or coexisting α-synuclein pathology	Arnulf et al. (2005) and Uchikado et al. (2006)
AD	Rare	Usually associated with mixed Lewy body pathology rather than isolated AD	Pure Alzheimer’s pathology appears unlikely to produce RBD independently	Boeve et al. (2013)
CBD	Rare	Occasional reports of RSWA or clinically manifest RBD	Evidence remains limited and inconclusive	Kimura et al. (1997) and Cullinane et al. (2022)
Pick’s disease	Very rare / not convincingly demonstrated	No consistent evidence of clinically manifest RBD	Current evidence does not support a significant association	Boeve et al. (2007)
Pallido-ponto-nigral degeneration	Not demonstrated	REM sleep atonia is generally preserved despite neurodegeneration	Suggests that selective disruption of REM-atonia circuits, rather than generalized neurodegeneration, is required for RBD	Boeve et al. (2006)

PD, Parkinson’s disease; MSA, multiple system atrophy; DLB Dementia with Lewy bodies; PAF, Pure autonomic failure; PSP, Progressive supranuclear palsy; AD, Alzheimer’s disease; CBD, Corticobasal degeneration.

These insights reinforce the role of RBD as a highly specific clinical biomarker of synuclein-driven neurodegeneration. At the same time, the presence of RBD across diverse conditions underscores its origin in distributed neural networks rather than a single pathological substrate. For clinicians, this dual perspective is essential: RBD should prompt evaluation for underlying synucleinopathy, while also considering alternative or coexisting mechanisms. For neuroscientists, RBD offers a unique model to study early network dysfunction in neurodegenerative disease, with direct implications for biomarker development and therapeutic intervention.

## 11 RBD as an early stage in the development of neurodegenerative disorders

A defining feature of RBD is its temporal relationship with neurodegenerative disease. In a substantial proportion of cases, isolated RBD precedes the onset of overt neurological symptoms by several years, and in some individuals by decades. Early cohort studies reported that RBD symptoms preceded Parkinson’s disease in approximately half of patients, with intervals ranging from 1 to 30 years. Longitudinal follow-up further demonstrated a progressive increase in conversion rates, with up to 65% of patients initially diagnosed with RBD developing a parkinsonian syndrome over extended observation periods (Olson et al., 2000; Schenck et al., 2013). More recent multicenter studies have reinforced these findings, showing annual phenoconversion rates of 6–8% and cumulative risks exceeding 80% over long-term follow-up (Postuma et al., 2019). These observations firmly establish RBD as one of the most robust clinical markers of prodromal neurodegeneration.

A compelling mechanistic framework for this temporal sequence is provided by the staging model of Parkinson’s disease proposed by Braak and colleagues. In early stages of the disease—before the emergence of motor symptoms—α-synuclein pathology is predominantly confined to lower brainstem and olfactory structures, including the dorsal motor nucleus of the vagus, the lower raphe nuclei, the gigantocellular reticular formation, and the locus coeruleus–subcoeruleus complex. These regions are critically involved in the regulation of REM sleep atonia. Their early involvement offers a biologically plausible explanation for the emergence of RBD as an initial clinical manifestation, preceding the degeneration of the substantia nigra and the onset of parkinsonism (Braak et al., 2003; Boeve et al., 2007; Postuma et al., 2019).

The hypothesis that isolated RBD represents an early stage of neurodegenerative disease is further supported by converging evidence from neurophysiology, imaging, cognition, and autonomic function in patients without overt neurological deficits. These findings suggest that RBD is not an isolated sleep disorder but rather part of a broader prodromal syndrome characterized by multisystem involvement (Miglis et al., 2021).

### 11.1 Electroencephalographic abnormalities

Quantitative electroencephalography (EEG) studies have identified slowing of waking cortical activity in patients with RBD, particularly increased theta power across frontal, temporal, and occipital regions (Fantini et al., 2003). This pattern is consistent with early cortical dysfunction and has been associated with an increased risk of cognitive impairment (Iranzo et al., 2010a). Similar EEG signatures have been reported in Parkinson’s disease and dementia with Lewy bodies, supporting a shared neurophysiological substrate. Importantly, in Parkinson’s disease, EEG slowing appears more pronounced in patients with coexisting RBD, suggesting that RBD identifies a subgroup with more extensive or diffuse neurodegeneration (Gagnon et al., 2004). Alterations in sleep architecture, including increased delta activity during slow-wave sleep, have also been described, although their functional significance remains to be fully elucidated (Massicotte-Marquez et al., 2005).

More recent studies have extended these observations from descriptive EEG slowing to predictive neurophysiological modelling. EEG-based machine learning models using baseline resting-state features, including spectral power, connectivity, and entropy measures, have been used to predict both phenoconversion timing and α-synucleinopathy subtype in isolated RBD. In a multicenter cohort, a random survival forest model predicted phenoconversion time with a concordance index of 0.775, whereas a K-nearest neighbor model predicted phenoconversion subtype with an area under the curve of 0.901; EEG slowing was an important feature in both models (Jeong et al., 2024).

Complementary evidence comes from EEG spectro-spatial covariance analysis. In patients with isolated RBD, converters showed higher disease-related beta-band pattern scores than nonconverters, and distinct EEG trajectories were observed among those converting to Parkinson’s disease, dementia with Lewy bodies, and those remaining nonconverted (Park et al., 2024). These findings suggest that quantitative EEG may contribute not only to global risk prediction but also to subtype-specific phenoconversion modelling, although larger multicenter validation remains necessary before implementation in clinical practice.

### 11.2 Neuroimaging markers of prodromal progression

Neuroimaging studies provide some of the strongest evidence that isolated RBD reflects ongoing neurodegeneration before the emergence of overt motor or cognitive syndromes. Dopaminergic imaging remains among the most validated prognostic tools. DAT-SPECT and PET studies consistently show reduced striatal dopamine transporter or presynaptic dopaminergic function in subsets of patients with isolated RBD, and abnormal dopaminergic imaging predicts future phenoconversion (Iranzo et al., 2010b; Postuma et al., 2019). Recent work has refined this approach by focusing not only on the presence of dopaminergic loss but also on its spatial pattern. Whole-brain dopaminergic damage patterns derived from 11C-CFT PET predicted phenoconversion timing in individual patients with isolated RBD, with dopaminergic degeneration detectable several years before clinical conversion (Feng et al., 2023).

Quantitative REM sleep without atonia may also relate to dopaminergic progression. In a large prospective cohort, REM sleep without atonia metrics correlated with DAT-SPECT binding ratios, suggesting that RSWA severity may reflect disease stage within prodromal α-synucleinopathy (Kunz et al., 2023). More recently, continuous quantification of REM sleep without atonia using cumulative RWA percentage outperformed conventional epoch-based methods for predicting phenoconversion, with an area under the curve of 0.89 and higher phenoconversion risk associated with greater cumulative RWA burden (Noh et al., 2025).

Metabolic imaging provides complementary information. Serial 18F-FDG PET studies indicate that expression of the Parkinson’s disease-related metabolic pattern increases over time in isolated RBD and rises more prominently in phenoconverters than in nonconverters, suggesting that longitudinal metabolic network measures may track prodromal disease progression (Carli et al., 2025). Functional MRI studies similarly support a network-level model of RBD. A systematic review of fMRI studies found functional alterations in isolated RBD across brainstem nuclei, basal ganglia, frontal and occipital regions, and whole-brain network measures; longitudinal functional connectivity changes may also track progression (Churchill et al., 2024).

Non-dopaminergic PET markers further support a multisystem model. Cholinergic PET imaging with 11C-donepezil showed that lower parietal cortical uptake was associated with a markedly higher rate of phenoconversion, while 18F-DOPA uptake abnormalities also predicted conversion (Terkelsen et al., 2024). In addition, microglial PET imaging suggests that neuroinflammatory activation may accompany progression of nigrostriatal dysfunction. Patients with increased baseline nigral microglial activation showed greater subsequent reductions in 18F-DOPA uptake, although the small sample size precludes firm conclusions regarding imminent phenoconversion (Stær et al., 2024).

Advanced MRI markers are also emerging. Quantitative susceptibility mapping and diffusion tensor imaging have identified midbrain and cerebral peduncle abnormalities associated with future phenoconversion, with combined susceptibility and fractional anisotropy measures showing predictive accuracy similar to dopamine-transporter imaging in one longitudinal cohort (Varga et al., 2025). In a multicenter study, reduced DTI-ALPS index, a proposed MRI proxy of glymphatic function, was associated with future phenoconversion, particularly to Parkinson’s disease, suggesting that impaired brain clearance mechanisms may contribute to prodromal progression in some patients (Ayral et al., 2025).

### 11.3 Cognitive deficits

Neuropsychological studies have identified subtle but consistent cognitive impairments in patients with RBD, particularly affecting visuospatial abilities, executive function, and both verbal and non-verbal learning. These deficits closely resemble the cognitive profile observed in synucleinopathies, especially dementia with Lewy bodies. Although some studies have methodological limitations, including small sample sizes and lack of polysomnographic confirmation, the overall pattern suggests that cognitive dysfunction may emerge early in the disease course and may predict progression to dementia (Fereshtehnejad et al., 2017; Postuma et al., 2019).

### 11.4 Perceptual abnormalities

Sensory dysfunction is another hallmark of the prodromal phase. Patients with isolated RBD frequently exhibit impairments in olfactory identification and discrimination, as well as deficits in color vision. These abnormalities are well-established early markers of Parkinson’s disease and are thought to reflect involvement of olfactory pathways and visual processing networks. Importantly, olfactory dysfunction in RBD correlates with subtle motor abnormalities, suggesting a shared underlying neurodegenerative process. The presence of these deficits in the absence of clinically manifest parkinsonism further supports the concept of isolated RBD as an early stage of disease (Postuma et al., 2019).

Recent evidence suggests that repeated olfactory testing may refine risk stratification beyond a single baseline assessment. In a longitudinal cohort of isolated RBD patients, persistent hyposmia was associated with worse DAT-SPECT indices and greater motor progression, whereas persistent normosmia was associated with no phenoconversion over approximately 5 years of follow-up. Baseline hyposmia alone did not predict phenoconversion, but repeated hyposmia significantly increased risk (Dall’Antonia et al., 2025). These findings suggest that serial olfactory assessment may be a low-cost strategy for enriching high-risk cohorts and identifying individuals with lower short-term phenoconversion risk.

### 11.5 Autonomic dysfunction

Autonomic disturbances are also common in RBD and may precede the onset of motor symptoms. Up to two-thirds of patients report symptoms such as orthostatic hypotension, constipation, or urinary dysfunction. Physiological studies have demonstrated reduced heart rate variability during sleep, reflecting impaired autonomic regulation. In addition, attenuated cardiovascular and cortical responses to internal stimuli, such as periodic limb movements, have been observed. These findings parallel those seen in synucleinopathies and suggest early involvement of autonomic networks (Fantini et al., 2002; Postuma et al., 2010; McCarter and Howell, 2017; Postuma et al., 2019).

Cardiac sympathetic imaging and fluid biomarkers may help differentiate future phenoconversion subtype. In a prospective cohort, elevated plasma neurofilament light chain predicted conversion to multiple system atrophy, whereas reduced cardiac MIBG uptake predicted conversion to Lewy body disease, including Parkinson’s disease and dementia with Lewy bodies (Park et al., 2023). These findings are consistent with the view that autonomic denervation and axonal injury markers may provide complementary information to motor, cognitive, and dopaminergic biomarkers.

### 11.6 Molecular and tissue biomarkers of α-synuclein pathology

Recent advances have shifted biomarker research in isolated RBD from indirect markers of neurodegeneration toward direct *in vivo* detection of misfolded α-synuclein. CSF RT-QuIC studies have shown that misfolded α-synuclein can be detected in most patients with isolated RBD and that positivity is associated with a higher risk of future Parkinson’s disease or dementia with Lewy bodies. In a longitudinal cohort, CSF α-synuclein RT-QuIC was positive in 90% of patients with isolated RBD, and 31 of 32 individuals who later developed Parkinson’s disease or dementia with Lewy bodies were α-synuclein-positive at baseline (Iranzo et al., 2021).

More recent studies indicate that α-synuclein pathology can also be detected outside the CSF. RT-QuIC assessment of skin and CSF samples in patients with isolated RBD showed comparable diagnostic performance, with high specificity and substantial agreement between both tissues, supporting the potential use of either CSF or skin biopsy for biological confirmation of prodromal synucleinopathy (Iranzo et al., 2023). Dermal RT-QuIC has also shown high sensitivity for α-synuclein aggregation in isolated RBD, although specificity may vary depending on the method and comparator group (Kuzkina et al., 2023). Comparative studies suggest that skin phosphorylated α-synuclein immunofluorescence and α-synuclein seed amplification assays provide complementary information, because immunofluorescence identifies pathological deposits in peripheral nerves whereas seed amplification assays measure seeding activity (Liguori et al., 2023).

The clinical relevance of these assays is supported by meta-analytic evidence. A recent meta-analysis reported substantial α-synuclein test positivity in isolated RBD, with pooled positivity estimates of approximately 80% for CSF α-synuclein seed amplification assays, 74.8% for skin α-synuclein seed amplification assays, and 78.5% for skin phosphorylated α-synuclein; skin-based testing and phosphorylated α-synuclein detection showed particularly high specificity (Iftikhar et al., 2024). These findings support the use of α-synuclein assays to distinguish biologically defined prodromal synucleinopathy from secondary or non-synuclein RBD.

However, α-synuclein negativity should not be interpreted simplistically. In a large cohort of patients with isolated RBD, approximately one quarter were CSF α-synuclein-negative. These patients were younger, had less REM sleep electromyographic activity, more frequent normal DAT-SPECT, normosmia, major depressive disorder, and antidepressant use, and showed a lower rate of phenoconversion to Parkinson’s disease or dementia with Lewy bodies during follow-up (Farfán et al., 2026). This observation suggests that α-synuclein-negative RBD may include very early synucleinopathy, delayed phenoconversion, or non-synucleinopathy-related phenotypes, with important implications for clinical counselling and neuroprotective trial enrichment.

Fluid biomarkers may also provide scalable measures of early neurodegeneration beyond α-synuclein detection. Plasma NfL and GFAP were higher in isolated RBD than in healthy controls, whereas pTau181 levels were similar, suggesting neuronal and glial injury without clear evidence of Alzheimer-type tau pathology at this stage (Liguori et al., 2025). However, these blood-based markers currently require longitudinal validation before they can be used for individualized prediction of phenoconversion.

### 11.7 Synthesis and implications

Taken together, these converging lines of clinical, neurophysiological, imaging, cognitive, sensory, autonomic, molecular, and fluid-biomarker evidence support a multidimensional model in which RBD represents an early manifestation of multisystem neurodegeneration. Recent evidence further refines this framework by showing that isolated RBD can be stratified according to converging abnormalities across sleep physiology, α-synuclein biology, dopaminergic and non-dopaminergic imaging, EEG, cognition, olfaction, autonomic function, and fluid biomarkers. The most important recent advance is the incorporation of direct α-synuclein assays, which allow biological confirmation of prodromal synucleinopathy in many patients, while also identifying α-synuclein-negative subgroups with lower or delayed phenoconversion risk (Iranzo et al., 2021; Iranzo et al., 2023; Muñoz-Lopetegi et al., 2024; Farfán et al., 2026) At the same time, quantitative RSWA, EEG-based models, PET/MRI markers, olfactory trajectories, and autonomic and fluid biomarkers provide complementary information about disease stage, progression rate, and likely clinical phenotype (Kunz et al., 2023; Jeong et al., 2024; Park et al., 2024; Dall’Antonia et al., 2025; Noh et al., 2025). The principal biomarker domains, recent findings, clinical significance, and limitations of these approaches are summarized in Table 2. A proposed framework for integrating these complementary biomarker domains into an individualized assessment of phenoconversion risk is shown in Figure 2.

**Table 2:** Multimodal biomarkers for risk stratification in REM sleep behavior disorder.

Biomarker domain	Principal findings in RBD	Clinical significance	Main limitations	References
vPSG and quantitative RSWA	RSWA remains the diagnostic hallmark of RBD. Quantitative RSWA metrics correlate with DAT-binding ratios, and continuous cumulative RWA percentage may improve prediction of phenoconversion.	Gold standard for diagnosis; quantitative RSWA may serve as a progression marker and trial-enrichment variable.	Scoring methods vary across centers; thresholds require harmonization; RSWA alone is insufficient for individualized prediction.	Cesari et al. (2022), Kunz et al. (2023), and Noh et al. (2025)
α-Synuclein seed amplification assays / RT-QuIC	Misfolded α-synuclein is detected in most patients with isolated RBD using CSF or skin RT-QuIC/SAA. CSF positivity predicts higher risk of PD/DLB conversion.	Provides biological confirmation of prodromal synucleinopathy and may enrich neuroprotective trials targeting α-synuclein.	Not all patients are positive; assay platforms, tissues, and thresholds vary; α-synuclein-negative RBD requires careful interpretation.	Iranzo et al. (2021), Iranzo et al. (2023), Siderowf et al. (2023), and Farfán et al. (2026)
Skin phosphorylated α-synuclein / dermal RT-QuIC	Skin biopsy can detect peripheral α-synuclein pathology. Dermal RT-QuIC shows high sensitivity, while phosphorylated α-synuclein immunofluorescence may provide high specificity.	Less invasive than some CNS-based approaches; useful for biological confirmation and differentiation from secondary RBD.	Diagnostic performance differs between techniques; skin site, biopsy processing, and comparator groups influence accuracy.	Kuzkina et al. (2023), Liguori et al. (2023), and Iftikhar et al. (2024)
CSF multimarker profile	CSF α-synuclein RT-QuIC positivity, mild cognitive impairment, and abnormal DAT-SPECT independently predict conversion to PD/DLB. AD biomarkers and NfL identify additional co-pathology or injury subgroups.	Supports integrated biological staging and may help distinguish risk for PD versus DLB.	Lumbar puncture may limit scalability; longer follow-up is needed to define the prognostic value of AD biomarkers and NfL.	Muñoz-Lopetegi et al. (2024)
Blood-based biomarkers	Plasma NfL and GFAP are increased in isolated RBD compared with controls, while pTau181 may remain similar to controls. Elevated NfL may predict conversion to MSA in selected cohorts.	Scalable and minimally invasive markers of neuronal/glial injury; potentially useful for repeated monitoring.	Limited specificity; longitudinal predictive thresholds remain uncertain.	Park et al. (2023) and Liguori et al. (2025)
Nigrostriatal dopaminergic imaging	DAT-SPECT/PET and 18F-DOPA PET reveal early presynaptic dopaminergic dysfunction. Whole-brain dopaminergic damage patterns may predict phenoconversion timing.	One of the strongest predictors of phenoconversion and useful for disease-stage estimation.	Cost, radiation exposure, limited availability, and overlap between prodromal and manifest disease stages.	Iranzo et al. (2010b), Postuma et al. (2019), Feng et al. (2023), and Terkelsen et al. (2024)
Metabolic PET / FDG-PET network patterns	Parkinson’s disease-related metabolic pattern expression increases over time in isolated RBD and is higher in converters than nonconverters.	May track prodromal progression and provide a network-level outcome marker.	Small longitudinal cohorts; radiation exposure; requires specialized analysis.	Carli et al. (2025)
Cholinergic PET	Lower cortical 11C-donepezil uptake, particularly in parietal cortex, is associated with impending phenoconversion.	Supports non-dopaminergic network involvement and may help identify short-term converters.	Small cohorts; limited tracer availability; requires replication.	Terkelsen et al. (2024)
Microglial PET / neuroinflammation	Increased nigral microglial activation is associated with greater subsequent decline in nigrostriatal dopaminergic function.	Suggests neuroinflammation may participate in early progression and may represent a therapeutic target.	Not yet established as an imminent phenoconversion marker; small sample size.	Stær et al. (2024)
Structural, diffusion, susceptibility, and glymphatic MRI	QSM/DTI markers in the cerebral peduncle predict phenoconversion with accuracy similar to DAT imaging in one cohort; reduced DTI-ALPS index is associated with future conversion, particularly to PD.	Noninvasive markers of brainstem, white-matter, iron-related, or glymphatic dysfunction.	Methods are heterogeneous; biomarkers are not yet standardized or clinically validated.	Ayral et al. (2025) and Varga et al. (2025)
Functional MRI	fMRI studies show alterations in brainstem nuclei, basal ganglia, frontal and occipital regions, and whole-brain networks in isolated RBD and PD-RBD.	Supports a distributed network model and may help develop noninvasive progression markers.	Cross-study heterogeneity; limited longitudinal validation.	Churchill et al. (2024)
EEG and EEG-based machine learning	EEG slowing, altered connectivity, spectro-spatial covariance patterns, and machine-learning models predict phenoconversion time and subtype.	Accessible, noninvasive tool for risk modelling and possible subtype prediction.	Analytical pipelines vary; needs larger multicenter validation.	Jeong et al. (2024) and Park et al. (2024)
Cognitive assessment	Executive, visuospatial, memory, and attention deficits may indicate early cortical involvement; MCI independently predicts conversion in CSF biomarker cohorts.	Useful for identifying patients at higher risk of DLB or cognitive phenoconversion.	Influenced by age, education, comorbidities, and test selection.	Gagnon et al. (2009), Postuma et al. (2019), and Muñoz-Lopetegi et al. (2024)
Olfactory testing	Persistent hyposmia, rather than baseline hyposmia alone, improves risk stratification; persistent normosmia may identify a lower-risk subgroup.	Low-cost, scalable marker for serial monitoring and trial enrichment.	Low disease specificity; affected by nasal disease, infection, smoking, medications, and other factors.	Postuma et al. (2011) and Dall’Antonia et al. (2025)
Autonomic assessment and cardiac MIBG	Autonomic symptoms are common. Reduced cardiac MIBG uptake may predict conversion to Lewy body disease, while elevated plasma NfL may point toward future MSA in some cohorts.	Helps identify multisystem involvement and may contribute to subtype prediction.	Autonomic symptoms are nonspecific; MIBG availability varies; cardiac comorbidities and medications may confound interpretation.	Postuma et al. (2010) and Park et al. (2023)
Multimodal biomarker integration	Combining sleep physiology, α-synuclein assays, imaging, EEG, cognition, olfaction, autonomic testing, and fluid biomarkers provides a more complete risk profile than any single marker.	Supports precision risk stratification, biological trial enrichment, and longitudinal monitoring.	Requires standardization, cost-effectiveness analysis, and prospective validation across diverse cohorts.	Miglis et al. (2021), Siderowf et al. (2023), Muñoz-Lopetegi et al. (2024), and Farfán et al. (2026)

EEG, electroencephalography; vPSG, video-polysomnography; RSWA, REM sleep without atonia.

**Figure 2:**
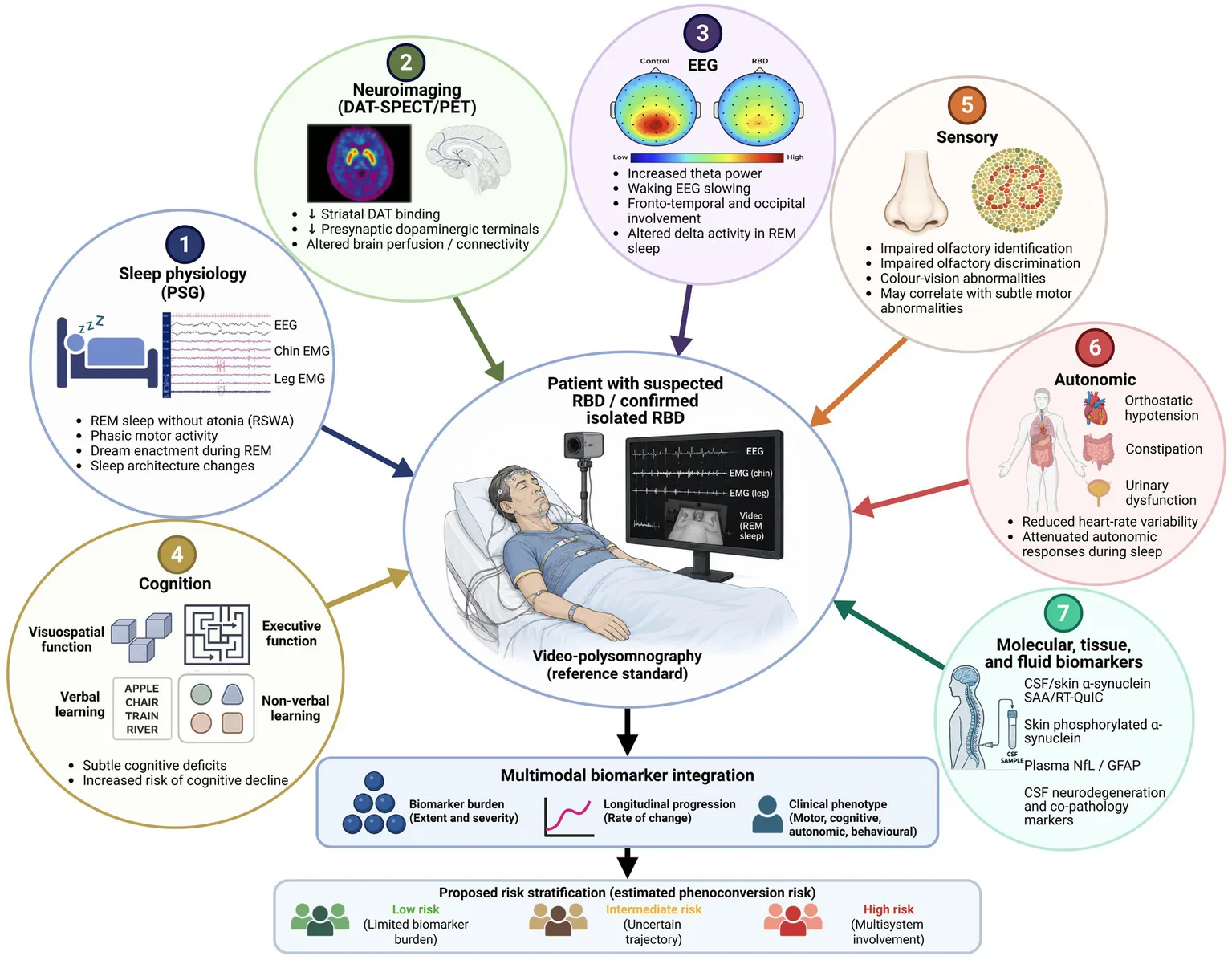
Multimodal biomarker framework for risk stratification in REM sleep behavior disorder. The figure illustrates how complementary biomarker domains may be integrated to support clinical assessment and longitudinal follow-up in patients with suspected or confirmed RBD, particularly isolated RBD. Video-polysomnography, the current diagnostic reference standard, documents REM sleep without atonia (RSWA) and may capture abnormal REM-related motor behaviors. Quantitative sleep-physiology measures derived from polysomnography can be considered alongside neuroimaging, electroencephalography (EEG), cognitive assessment, sensory evaluation—including olfactory and color-vision testing—autonomic-function assessment, and molecular, tissue, and fluid biomarkers. These latter biomarkers may include CSF or skin α-synuclein seed amplification assays/RT-QuIC, skin phosphorylated α-synuclein, plasma neurofilament light chain and glial fibrillary acidic protein, and CSF markers of neurodegeneration or co-pathology. Together, these domains provide complementary information on sleep physiology, motor-system integrity, molecular pathology, cognition, sensory function, autonomic involvement, and other features relevant to prodromal neurodegeneration. Their integration may help estimate overall biomarker burden, monitor longitudinal change, and characterize clinical phenotype, thereby supporting proposed low-, intermediate-, or high-risk categories for future phenoconversion. These risk categories are conceptual and should be interpreted as probabilistic rather than deterministic; they require validation in prospective longitudinal cohorts before routine clinical application. The framework illustrates a possible transition from single-modality diagnosis toward longitudinal, biomarker-informed approaches to assessment and follow-up. Image created by the authors using BioRender.

## 12 A network-based hypothesis for RBD: from REM atonia failure to systems-level neurodegeneration

The convergence of clinical, neurophysiological, imaging, biomarker, and neuropathological evidence supports a unifying hypothesis in which RBD may represent one of the earliest clinically detectable manifestations of a systems-level neurodegenerative process. This process is characterized by early vulnerability of brainstem circuits that regulate REM sleep atonia, with variable involvement of subcortical, autonomic, limbic, and cortical networks over time (Dauvilliers et al., 2018; Berg et al., 2021). Within this framework, the network-based model proposed here should be understood as a conceptual synthesis of converging evidence rather than a fixed or universal staging sequence. Although current data strongly support the prodromal-neurodegeneration framework, the anatomical origin, temporal evolution, propagation pattern, and eventual clinical phenotype are heterogeneous across patients and remain incompletely defined.

This interpretation should be reconciled with the heterogeneity of prodromal synucleinopathy. A brainstem-predominant trajectory is particularly relevant to RBD because REM sleep atonia depends on pontomedullary circuits, and post-mortem and imaging studies support early involvement of structures implicated in REM motor control (Boeve et al., 2007; Peever and Fuller, 2017; Dauvilliers et al., 2018; Mayà et al., 2024). However, this does not imply that all patients follow a uniform brainstem-to-cortex sequence. Body-first and brain-first models propose different initial sites and directions of pathological progression, and RBD may be more closely associated with body-first or brainstem-involved phenotypes in many patients, whereas other trajectories may begin or become clinically relevant through olfactory, autonomic, limbic, cortical, or nigrostriatal pathways (Berg et al., 2021; Miglis et al., 2021). Therefore, the brainstem-to-cortex model is best understood as one plausible disease trajectory within a broader set of prodromal synucleinopathy patterns, rather than as a universal staging sequence.

At the core of this hypothesis lies the concept of REM motor gating failure. Under physiological conditions, REM sleep is defined by a dissociation between cortical activation and peripheral motor inhibition. This state is actively maintained by a distributed network centered in the pontomedullary brainstem, including the subcoeruleus region and medullary reticular formation, which suppress spinal motor neuron activity. In RBD, this inhibitory system becomes unstable, allowing motor output to escape during REM sleep (Peever and Fuller, 2017). Importantly, this failure is not binary but exists along a continuum, ranging from isolated RSWA to overt dream enactment behaviors (Cesari et al., 2022).

We propose that this instability reflects an early network-level imbalance between excitatory and inhibitory control within REM circuits, driven by subtle dysfunction across multiple neurotransmitter systems, including noradrenergic, serotonergic, cholinergic, and dopaminergic pathways. Rather than a single lesion or focal pathology, RBD emerges from a state of reduced resilience within these interconnected systems (Dauvilliers et al., 2018). This framework is consistent with pharmacological observations showing partial dissociation between tonic atonia and phasic motor suppression (Lapierre and Montplaisir, 1992), as well as with imaging studies demonstrating early dopaminergic deficits in the absence of motor symptoms (Iranzo et al., 2010b).

A key implication of this conceptual model is that, in many patients, RBD may represent one of the first clinically accessible manifestations of a broader neurodegenerative process involving REM sleep regulatory, autonomic, dopaminergic, limbic, and cortical networks. Neuropathological studies indicate that the brainstem nuclei implicated in REM atonia are among the earliest sites of pathology in α-synucleinopathies (Braak et al., 2003). From this initial locus, pathological processes, potentially involving misfolded protein propagation, impaired proteostasis, and neuroinflammatory responses, extend to the substantia nigra, limbic structures, and eventually cortical regions (Braak et al., 2003; Iranzo et al., 2021; Stær et al., 2024). This hierarchical spread mirrors the clinical trajectory observed in longitudinal studies, where isolated RBD precedes motor, cognitive, and autonomic manifestations by several years (Postuma et al., 2019). Misfolded α-synuclein can already be detected in most patients with isolated RBD, and PET evidence indicates that microglial activation may accompany subsequent nigrostriatal deterioration.

This biological interpretation is now supported by convergent CSF, skin, and post-mortem evidence. Seed amplification assays detect misfolded α-synuclein in most patients with isolated RBD, while post-mortem data show α-synuclein pathology in REM-atonia regulatory structures, including the subcoeruleus nucleus and gigantocellular reticular nucleus (Iranzo et al., 2021; Iranzo et al., 2023; Mayà et al., 2024). Nevertheless, the presence of α-synuclein-negative RBD, variable dopaminergic involvement, frequent co-pathology, and differing trajectories toward Parkinson’s disease, dementia with Lewy bodies, or multiple system atrophy indicate that RBD should be conceptualized as a biologically heterogeneous prodromal syndrome rather than a uniform linear stage (Mayà et al., 2024; Farfán et al., 2026).

Within this framework, the heterogeneity of RBD becomes biologically meaningful. Variability in clinical expression, including the presence or absence of overt behaviors, the rate of progression, and the development of specific neurodegenerative phenotypes, may reflect differences in the topography, burden, and propagation dynamics of the underlying pathology (Berg et al., 2021). For example, early involvement of limbic circuits may account for the emotional and dream-related features of RBD, whereas extension to dopaminergic pathways predicts the emergence of parkinsonism. Similarly, cortical involvement may underlie the cognitive deficits observed in a subset of patients (Dauvilliers et al., 2018). Current models distinguish body-first and brain-first patterns and therefore support variation in pathological topography and progression rather than a universal linear sequence.

Importantly, this hypothesis integrates RBD into a broader concept of prodromal neurodegenerative syndromes, in which multiple systems are affected before the onset of classical diagnostic features. The co-occurrence of EEG slowing, olfactory dysfunction, visuospatial deficits, and autonomic abnormalities in patients with RBD supports this view, suggesting that RBD is one component of a multisystem disorder rather than an isolated entity (Miglis et al., 2021).

From a translational perspective, this model has several critical implications. First, it positions RSWA and RBD as physiological biomarkers of early network dysfunction, with potential utility in identifying individuals at high risk of neurodegenerative disease (Miglis et al., 2021; Cesari et al., 2022). Second, it highlights the need for multimodal risk stratification, integrating polysomnography with neuroimaging, neurophysiology, and clinical phenotyping to capture the full spectrum of early disease (Miglis et al., 2021). Third, it suggests that effective intervention will require targeting network stability and proteostatic mechanisms, rather than focusing solely on symptomatic control of nocturnal behaviors (Schenck et al., 2013; Iranzo et al., 2021). Misfolded α-synuclein assays may be particularly useful for enriching future neuroprotective trials with biologically defined participants.

This hypothesis demands a redefinition of therapeutic goals. Current treatments, such as clonazepam and melatonin, reduce behavioral manifestations but do not address the underlying pathophysiology (Howell et al., 2023b). In contrast, a network-based approach would prioritize early intervention strategies aimed at stabilizing REM circuitry, modulating neurotransmitter balance, and preventing pathological spread. Such strategies could transform RBD from a marker of inevitable progression into a window for disease modification (Schenck et al., 2013).

Several caveats must be emphasized. First, not all patients with isolated RBD follow the same trajectory, and phenoconversion may lead to Parkinson’s disease, dementia with Lewy bodies, multiple system atrophy, or mixed phenotypes. Second, α-synuclein-positive and α-synuclein-negative RBD may represent biologically distinct or temporally different states. Third, coexisting Alzheimer-type, vascular, inflammatory, or age-related pathologies may influence clinical expression, especially cognitive outcomes. Finally, current biomarker models remain probabilistic rather than deterministic; they can refine group-level risk but cannot yet provide definitive individualized prediction. Therefore, the proposed network-based hypothesis should be regarded as a framework for organizing current evidence and generating testable predictions, rather than as a settled model of disease progression.

In summary, RBD can be conceptualized as a sentinel event in the trajectory of neurodegeneration, reflecting early failure of REM sleep regulatory networks and heralding the subsequent involvement of broader brain systems (Dauvilliers et al., 2018; Miglis et al., 2021). The proposed disease stages and factors contributing to heterogeneous clinical trajectories are summarized in Figure 3. This perspective provides a coherent framework to integrate clinical observations with mechanistic insights and offers a foundation for the development of next-generation diagnostic and therapeutic approaches (Miglis et al., 2021).

**Figure 3:**
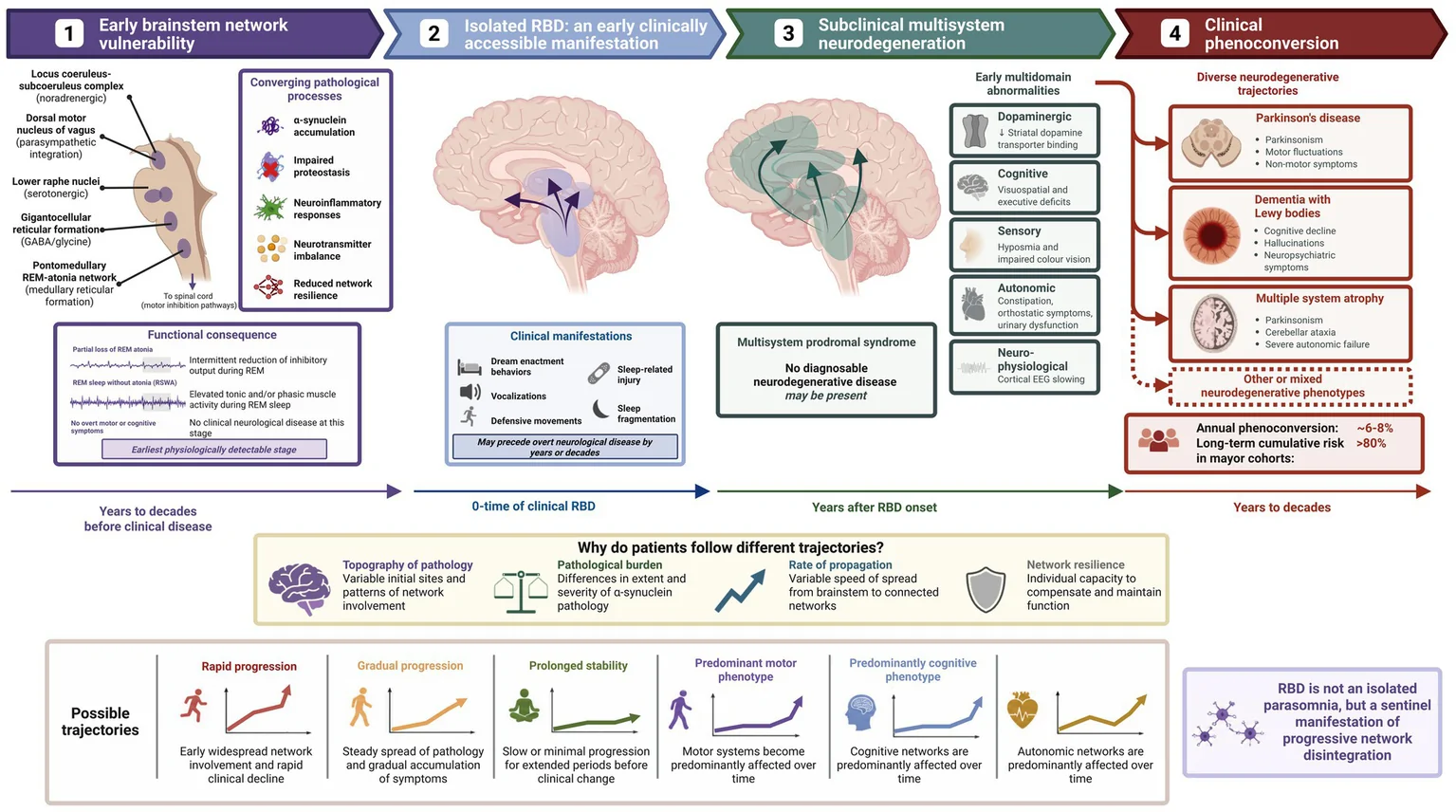
Proposed network-based model of RBD as an early clinically accessible manifestation of neurodegenerative vulnerability. The figure presents a conceptual systems-level model linking dysfunction of REM sleep regulatory networks with the possible emergence of clinically defined neurodegenerative disease over time. Stage 1 depicts subclinical dysfunction of brainstem nuclei involved in REM sleep atonia, which may be accompanied by α-synuclein accumulation, impaired proteostasis, neuroinflammatory responses, neurotransmitter imbalance, and reduced network resilience. Partial loss of REM atonia or isolated REM sleep without atonia may be physiologically detectable before overt dream-enactment behavior or other neurological manifestations. Stage 2 represents clinically manifest isolated RBD, characterized by dream-enactment behaviors, vocalizations, defensive movements, sleep fragmentation, and possible sleep-related injury in the absence of a diagnosable neurodegenerative disorder or another clear secondary cause. Stage 3 illustrates a multisystem prodromal phase, during which abnormalities may become detectable across dopaminergic, cognitive, sensory, autonomic, and neurophysiological domains. Stage 4 represents possible clinical phenoconversion to Parkinson’s disease, dementia with Lewy bodies, multiple system atrophy, or other mixed neurodegenerative phenotypes. Reported annual phenoconversion rates in isolated RBD are approximately 6–8%, with cumulative risks exceeding 80% in some long-term cohorts; these values represent population-level estimates and should not be interpreted as deterministic predictions for individual patients. The lower panels summarize factors that may contribute to heterogeneous disease trajectories, including pathological topography and burden, propagation rate, comorbid pathology, and network resilience. Representative trajectories include rapid or gradual progression, prolonged clinical stability, and predominantly motor, cognitive, or autonomic phenotypes. The proposed stages and trajectories are conceptual, not necessarily linear or universal, and require further longitudinal and biological validation. Image created by the authors using BioRender.

## 13 Discussion

The reframing of RBD as an early manifestation of neurodegeneration represents a major conceptual advance, yet its translation into clinical practice and mechanistic understanding remains incomplete. The current evidence base, although robust in demonstrating associations and longitudinal risk, exposes important gaps that must be addressed to move the field from observation to intervention (Dauvilliers et al., 2018; Miglis et al., 2021).

Although longitudinal studies have consistently shown that isolated RBD is associated with a high risk of future α-synucleinopathy, reported phenoconversion rates vary substantially across cohorts. This variability likely reflects differences in recruitment setting, follow-up duration, age at diagnosis, sex distribution, diagnostic criteria, polysomnographic protocols, medication exposure, comorbidities, and the definition of phenoconversion endpoints. Many cohorts are derived from tertiary sleep or movement-disorders centers, which may enrich samples for individuals with more severe dream-enactment behaviors, higher injury risk, or additional prodromal features. Consequently, estimates derived from these cohorts may not be directly generalizable to community-detected RBD, milder cases, younger patients, women, or individuals with antidepressant-associated or otherwise atypical presentations.

Several biomarker studies are also limited by small sample sizes, single-center designs, incomplete follow-up, and differences in acquisition and analysis methods. This is particularly relevant for newer approaches such as quantitative RSWA metrics, α-synuclein seed amplification assays, skin biopsy markers, EEG-based machine learning, advanced PET, functional MRI, diffusion MRI, glymphatic imaging, and plasma biomarkers. Although these methods are promising, many still require independent replication, harmonized thresholds, longitudinal validation, and assessment of clinical utility before they can be adopted as individualized prognostic tools. Thus, while the available evidence strongly supports RBD as a major prodromal marker of α-synucleinopathy, individual trajectories remain heterogeneous: some patients convert rapidly, others remain stable for many years, and a subset may represent very early, delayed, mixed-pathology, medication-associated, or non-synucleinopathy-related phenotypes.

A first challenge lies in the persistence of traditional nosological boundaries. RBD continues to be classified as a parasomnia, a categorization that fails to reflect its role as a systems-level disorder linked to neurodegenerative processes. This conceptual limitation has practical consequences, as it constrains clinical thinking and delays the integration of RBD into neurological diagnostic frameworks. A redefinition that situates RBD within the spectrum of prodromal neurodegenerative syndromes is necessary to align classification with biological reality (Högl et al., 2022).

Closely related is the challenge of early detection, particularly in individuals with subclinical manifestations such as isolated RSWA. Although polysomnography can identify these early abnormalities (Cesari et al., 2022), their prognostic significance remains uncertain. It is not yet clear whether all individuals with RSWA will progress to clinically manifest RBD or neurodegenerative disease, or whether this group represents a heterogeneous population with variable trajectories. Longitudinal studies with rigorous phenotyping are therefore essential to clarify the natural history of these early states (Stefani et al., 2015). The International RBD Study Group guidelines recognize isolated RSWA as a possible prodromal manifestation but emphasize that its natural history and predictive significance are not yet sufficiently established.

The diagnostic process itself also requires refinement. While polysomnography is the gold standard, variability in acquisition protocols, scoring criteria, and interpretation limits its reproducibility across centers. The absence of standardized quantitative thresholds for RSWA introduces uncertainty and reduces comparability between studies (Cesari et al., 2022). The development of harmonized methodologies, potentially supported by automated and machine learning–based analysis, will be critical to enhance diagnostic precision and scalability (Cooray et al., 2019).

Another important limitation is the challenge of differential diagnosis. RBD shares clinical features with a range of conditions, including obstructive sleep apnea, nocturnal epilepsy, and non-REM parasomnias, as well as pharmacologically induced and immune-mediated states. Distinguishing true RBD from these mimics requires not only technical expertise but also an integrated clinical approach that incorporates polysomnographic, neurological, and contextual information. Failure to do so risks both overdiagnosis and missed opportunities for early identification of neurodegenerative disease (Antelmi et al., 2021).

Beyond diagnosis, the marked heterogeneity of RBD presents a significant challenge. Patients differ in symptom severity, progression rate, associated biomarkers, and eventual clinical outcomes. Some individuals rapidly convert to Parkinson’s disease or dementia with Lewy bodies, whereas others remain stable for years (Postuma et al., 2019). This variability suggests underlying biological diversity, possibly reflecting differences in the distribution and propagation of pathology. Elucidating these subtypes through multimodal approaches will be essential for personalized prognosis and targeted intervention (Berg et al., 2021; Miglis et al., 2021). Longitudinal cohorts demonstrate substantial variation in phenoconversion timing, while contemporary subtype models propose different spatial and temporal patterns of neurodegenerative progression.

Recent data also highlight the importance of demographic and clinical context in interpreting phenoconversion risk. In a sex-balanced retrospective cohort, women were diagnosed with isolated RBD at a younger age than men, and crude conversion rates differed between groups; however, after adjustment for symptom duration, sex, antidepressant use, psychiatric comorbidity, chronic pain, and vascular comorbidity, age at diagnosis was the only independent predictor of phenoconversion (Alexandres et al., 2024). These findings suggest that sex differences, psychiatric comorbidity, antidepressant exposure, and age at diagnosis should be considered when interpreting individual risk, particularly in cohorts that include younger patients or women, but should not be overinterpreted as isolated predictors without multivariable adjustment.

These sources of variability have direct implications for prognostic counseling. Phenoconversion estimates derived from longitudinal cohorts are population-level probabilities and should not be communicated as deterministic predictions for individual patients. Although isolated RBD increases the likelihood of future α-synucleinopathy, available data cannot determine with certainty whether a given patient will convert, when phenoconversion will occur, or whether the eventual phenotype will be Parkinson’s disease, dementia with Lewy bodies, multiple system atrophy, or another clinical presentation. Counseling should therefore be individualized, staged, and preference-sensitive, taking into account the patient’s age, comorbidities, medication exposure, biomarker profile, psychological readiness, and desire for prognostic information (Postuma et al., 2019; Miglis et al., 2021). This is particularly important because no established treatment currently prevents phenoconversion. Accordingly, disclosure of risk should be balanced with practical clinical benefits, including injury prevention, safety counseling, treatment of dream enactment behaviors, review of potentially contributing medications, identification and management of treatable contributors such as comorbid sleep disorders, longitudinal monitoring for motor, cognitive, autonomic, and psychiatric symptoms, advance planning when appropriate, and discussion of research participation (Schenck et al., 2013; Howell et al., 2023a). In this context, biomarker-based risk stratification should be presented as a tool for refining follow-up, counseling, and research eligibility rather than as a definitive prediction of an individual patient’s clinical future.

At a mechanistic level, a major unresolved issue is the integration of multiple neurotransmitter systems into a coherent model. Although dopaminergic, noradrenergic, serotonergic, and cholinergic pathways are all implicated in REM sleep regulation, their relative contributions and interactions remain poorly defined. Current models describe a state of network instability, but lack the resolution needed to identify specific therapeutic targets. Advancing from descriptive to mechanistic frameworks will require interdisciplinary approaches that combine systems neuroscience, neurochemistry, and computational modelling (Peever and Fuller, 2017; Dauvilliers et al., 2018).

Equally important is the need to understand how local circuit dysfunction evolves into widespread neurodegeneration. The association between RBD and early brainstem pathology is well established, particularly in relation to α-synuclein deposition. However, the mechanisms by which pathology propagates to subcortical and cortical regions remain uncertain. Whether this process is driven by trans-synaptic spread, network vulnerability, or systemic factors such as inflammation and proteostatic failure is still debated. Resolving this question is central to developing disease-modifying strategies (Dauvilliers et al., 2018; Berg et al., 2021). Current models support considerable heterogeneity in the anatomical origin and progression of synucleinopathy rather than one universal brainstem-to-cortex sequence.

The integration of biomarkers represents another critical frontier. RBD is associated with abnormalities across multiple domains, including electroencephalography, dopaminergic imaging, cognition, olfaction, and autonomic function. Yet these markers are often studied in isolation, limiting their predictive value. A multimodal approach that combines these features into composite risk models is likely to provide more accurate stratification and improve the identification of individuals at highest risk of phenoconversion (Miglis et al., 2021).

Therapeutically, the field remains constrained by a reliance on symptomatic treatments. Clonazepam and melatonin reduce nocturnal behaviors but do not modify the underlying disease process (Howell et al., 2023b). This limitation reflects a broader gap in the development of interventions targeting early neurodegeneration. RBD cohorts offer a unique opportunity for clinical trials at a stage when neuronal dysfunction may still be reversible, yet this potential has not been fully realized. The identification of appropriate endpoints, biomarkers, and trial designs is therefore an urgent priority (Schenck et al., 2013; Miglis et al., 2021). Current systematic evidence supports symptomatic management but has not demonstrated prevention of phenoconversion or modification of the underlying α-synucleinopathy.

Finally, there is a translational challenge in implementing these insights into routine clinical care. Despite strong evidence linking isolated RBD to neurodegenerative disease, many patients remain undiagnosed or are managed without further neurological evaluation (Pujol et al., 2017; Teigen et al., 2021). There is a need for structured clinical pathways that incorporate screening, polysomnographic confirmation, biomarker assessment, and longitudinal follow-up. Such frameworks would facilitate early diagnosis, improve patient counselling, and create opportunities for intervention (Miglis et al., 2021; Teigen et al., 2021). A proposed biomarker-guided staging and precision-management pathway integrating these elements is presented in Figure 4.

**Figure 4:**
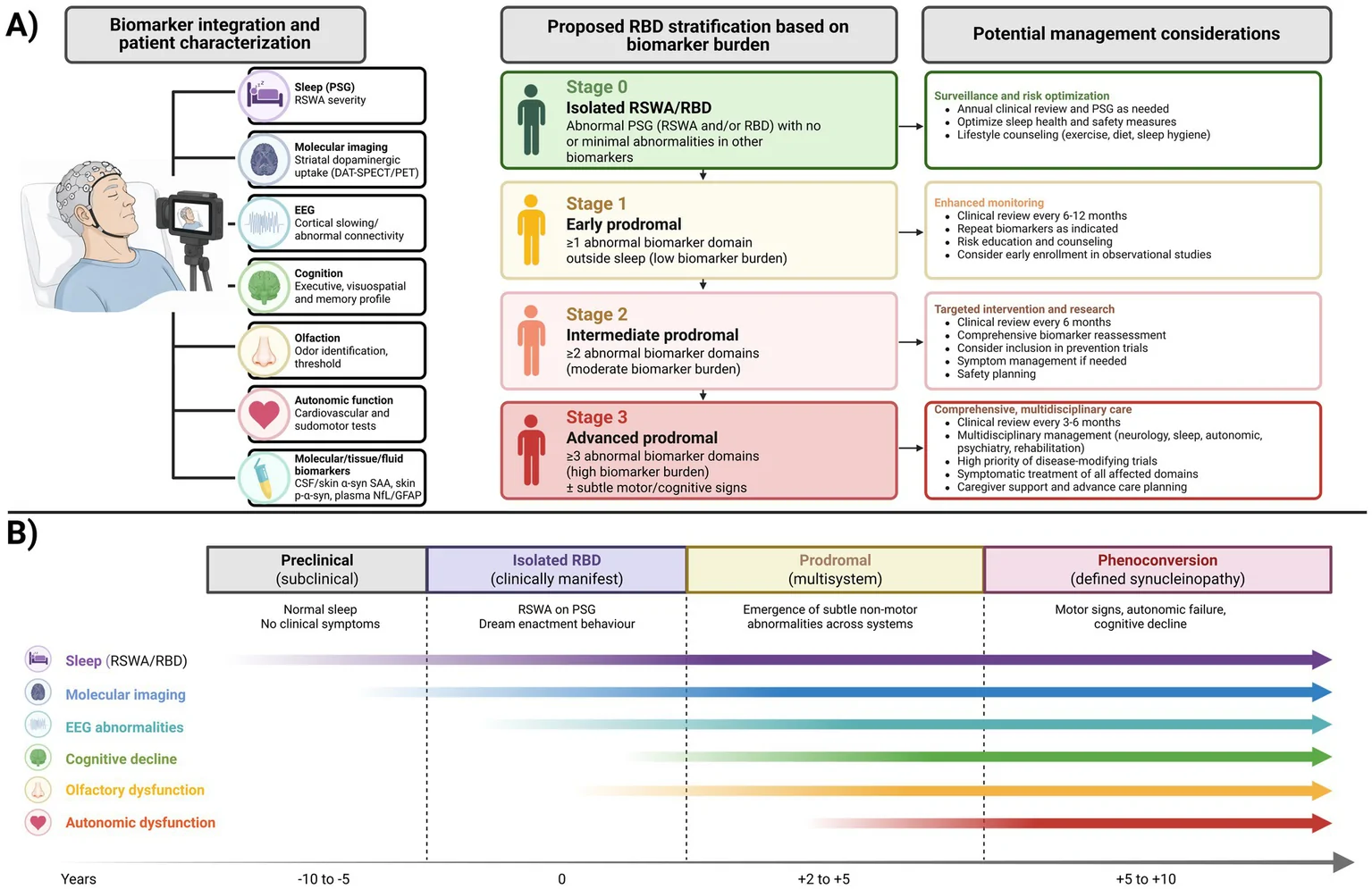
Proposed biomarker-informed framework for clinical assessment and longitudinal management in REM sleep behavior disorder. **(A)** Conceptual clinical framework illustrating how multimodal biomarkers may contribute to risk stratification and follow-up planning in individuals with isolated REM sleep without atonia (RSWA) or confirmed RBD, particularly isolated RBD. Following polysomnographic identification of RSWA or confirmation of RBD, abnormalities across sleep physiology, nigrostriatal molecular imaging, electroencephalography, cognition, olfaction, and autonomic function may be considered together to estimate overall biomarker burden. The proposed stages range from isolated RBD with few or no additional biomarker abnormalities to more extensive multisystem prodromal involvement. Increasing biomarker burden may support closer clinical surveillance, periodic biomarker reassessment, multidisciplinary evaluation, safety planning, and consideration of participation in observational studies or disease-modification trials when appropriate. The biomarker thresholds, surveillance intervals, and management approaches shown are illustrative and should not be interpreted as validated clinical guidelines. **(B)** Proposed temporal evolution and overlap of biomarker abnormalities across a possible neurodegenerative continuum, extending from a preclinical or subclinical phase through clinically manifest isolated RBD, multisystem prodromal disease, and possible phenoconversion to a defined synucleinopathy. Biomarker domains may emerge at different times, in different sequences, and with substantial overlap among individuals. The temporal intervals are approximate and are intended to illustrate possible trajectories rather than fixed disease stages. Overall, the framework emphasizes longitudinal, multimodal assessment as a complement to clinical evaluation and polysomnography, rather than reliance on isolated diagnostic tests. Image created by the authors using BioRender.

## 14 Conclusion

REM sleep behavior disorder has emerged as an important interface between sleep physiology and neurodegenerative disease. The evidence reviewed in this manuscript supports a model in which RBD reflects early dysfunction of REM sleep regulatory networks and, in many patients, precedes broader neurodegenerative pathology, particularly within the α-synucleinopathy spectrum.

RBD should therefore not be viewed solely as a disorder of abnormal nocturnal behavior, but also as a clinically relevant marker of increased neurodegenerative risk in appropriately characterized patients. At the same time, individual trajectories remain heterogeneous and are influenced by differences in pathology, comorbidities, biomarker profiles, and follow-up duration.

Future progress will depend on integrating polysomnography with molecular, neuroimaging, neurophysiological, autonomic, cognitive, and fluid biomarkers to refine risk stratification and monitor disease progression. In this context, RBD provides a useful model of early brain vulnerability and a potential platform for earlier, biologically informed therapeutic strategies.
